# Food–bacteria interplay: pathometabolism of emetic *Bacillus cereus*

**DOI:** 10.3389/fmicb.2015.00704

**Published:** 2015-07-14

**Authors:** Monika Ehling-Schulz, Elrike Frenzel, Michel Gohar

**Affiliations:** ^1^Functional Microbiology, Institute of Microbiology, Department of Pathobiology, University of Veterinary Medicine ViennaVienna, Austria; ^2^INRA, UMR1319 Micalis, AgroParistech – Domaine de Vilvert, Génétique Microbienne et EnvironnementJouy-en-Josas, France

**Keywords:** emetic toxin, cereulide, *Bacillus cereus*, foodborne intoxication, non-ribosomal peptide synthetase, pathometabolism

## Abstract

*Bacillus cereus* is a Gram-positive endospore forming bacterium known for its wide spectrum of phenotypic traits, enabling it to occupy diverse ecological niches. Although the population structure of *B. cereus* is highly dynamic and rather panmictic, production of the emetic *B. cereus* toxin cereulide is restricted to strains with specific genotypic traits, associated with distinct environmental habitats. Cereulide is an ionophoric dodecadepsipeptide that is produced non-ribosomally by an enzyme complex with an unusual modular structure, named cereulide synthetase (Ces non-ribosomal peptide synthetase). The *ces* gene locus is encoded on a mega virulence plasmid related to the *B. anthracis* toxin plasmid pXO1. Cereulide, a highly thermo- and pH- resistant molecule, is preformed in food, evokes vomiting a few hours after ingestion, and was shown to be the direct cause of gastroenteritis symptoms; occasionally it is implicated in severe clinical manifestations including acute liver failures. Control of toxin gene expression in emetic *B. cereus* involves central transcriptional regulators, such as CodY and AbrB, thereby inextricably linking toxin gene expression to life cycle phases and specific conditions, such as the nutrient supply encountered in food matrices. While in recent years considerable progress has been made in the molecular and biochemical characterization of cereulide toxin synthesis, far less is known about the embedment of toxin synthesis in the life cycle of *B. cereus.* Information about signals acting on toxin production in the food environment is lacking. We summarize the data available on the complex regulatory network controlling cereulide toxin synthesis, discuss the role of intrinsic and extrinsic factors acting on toxin biosynthesis in emetic *B. cereus* and stress how unraveling these processes can lead to the development of novel effective strategies to prevent toxin synthesis in the food production and processing chain.

## Introduction

*Bacillus cereus* is an ubiquitous Gram-positive, facultative anaerobic rod-shaped bacterium that is notorious due to the formation of highly heat, acid, UV, and desiccation resistant endospores. It is increasingly recognized as an opportunistic pathogen causing gastrointestinal as well as local and systemic infections (Bottone, 2010; Ehling-Schulz et al., 2011). Because of its broad spectrum of phenotypic traits, *B. cereus* can enter food production and processing chains at various points, confronting the food industry with several challenges. Beside its food spoilage potential, it bears a severe health risk for consumers. Several toxins have been described that may cause two types of food borne diseases. The non-hemolytic enterotoxin complex (NHE) and the hemolytic enterotoxin complex (HBL) as well as a variant of the single cytotoxin K have been linked to the diarrheal form of the disease, while the depsipeptide toxin cereulide has been shown to be the causative agent of the emetic form of the disease (Ehling-Schulz et al., 2004; Schoeni and Wong, 2005; Stenfors Arnesen et al., 2008). In the past, diagnostics were hampered by the lack of appropriate detection and identification systems rendering it impossible to estimate the true incidence of food borne diseases related to *B. cereus*. However, during the last decade several tools, such as multiplex PCR for toxin gene profiling, *in situ* monitoring of cereulide synthesis with luminescent reporter strains, or mass spectrometry-based methods for toxin quantitation in complex food matrices have been developed (Ehling-Schulz et al., 2006b; Bauer et al., 2010; Dommel et al., 2010; Stark et al., 2013), which facilitate and significantly improve *B. cereus* diagnostics (Ehling-Schulz and Messelhäusser, 2013). Indeed, the number of reported food poisonings related to *B. cereus* toxins has shown a steep increase from 2006 onward, and is still increasing (Anonymous, 2009, 2015). Yet, in most countries the reporting of *B. cereus*-induced gastroenteritis is mandatory only in foodborne outbreak cases, leading to an underestimation of this bacterial incidence on public health. The capacity for enterotoxin production is widely distributed within the *B. cereus* population. In contrast, emetic toxin production is restricted to strains with specific genotypic and phenotypic traits, although some diversification seems to be ongoing (Prüss et al., 1999; Ehling-Schulz et al., 2005a, 2011; Thorsen et al., 2006; Guinebretiere et al., 2008; Hoton et al., 2009).

Both forms of the disease are normally self-limiting but some more severe forms requiring hospitalization are occasionally reported (Lund et al., 2000; Fricker et al., 2007; Ehling-Schulz and Messelhäusser, 2012). Especially the emetic toxin cereulide is increasingly linked to severe clinical manifestations including acute liver failures and has also been reported as a causative agent of acute encephalopathy (Dierick et al., 2005; Posfay-Barbe et al., 2008; Ichikawa et al., 2010; Naranjo et al., 2011; Tschiedel et al., 2015). Recent results from *in vitro* tests showed a detrimental effect of cereulide on pancreatic beta cells, even in low concentrations. Since beta cell dysfunction and cell death plays a key role in pathophysiology of diabetes, these results render it possible that a long-termed uptake of cereulide-contaminated food could have some implications in diabetes (Vangoitsenhoven et al., 2014).

The diarrheal type of disease is thought to be the consequence of a foodborne infection with enterotoxic *B. cereus* and enterotoxin production after outgrowth of bacterial spores in the intestine, while the emetic syndrome is presumably the result of intoxication by cereulide, previously formed in food contaminated with emetic *B. cereus*. Thus, an understanding of the interplay of food environment and emetic *B. cereus* is of utmost importance to fully decipher mechanisms of pathogenicity of emetic *B. cereus* and to develop novel strategies for the prevention of cereulide toxin production in the food production and processing chain. While in recent years considerable progress has been made in the molecular and biochemical characterization of cereulide toxin synthesis, far less is known about the embedment of toxin synthesis in the life cycle of *B. cereus* and the primary ecological niches of emetic *B. cereus* are largely unknown.

## An Unusual Enzyme Complex for an Unusual Toxin: Architecture of the Genetic Locus for Cereulide Depsipeptide Toxin Production

### Intracellular Biochemical Factory for Cereulide Production

**FIGURE 1:**
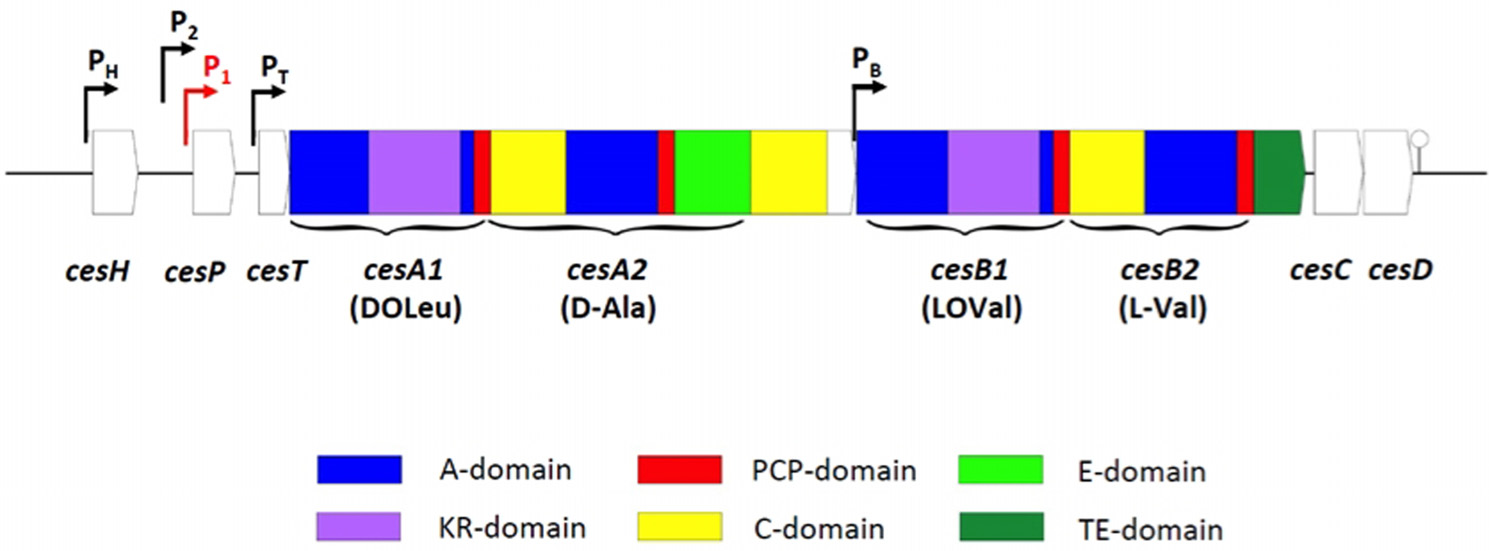
**Genetic architecture of the cereulide synthetase gene cluster (according to Ehling-Schulz et al., 2006a; Dommel et al., 2010).** Promoters are indicated by bent arrows. The main promoter driving the polycistronic transcription of the *ces* operon is highlighted in red. A hairpin indicates the terminator.

In contrast to the enterotoxin genes, which are ‘classical’ protein toxins produced *via* the ribosomal pathway, the emetic depsipeptide toxin cereulide [D-*O*-Leu-D-Ala-L-*O*-Val-L-Val]_3_ is produced by a non-ribosomal peptide synthetase (NRPS; Ehling-Schulz et al., 2005b). NRPS are large modular multienzyme complexes that act as *in vivo* biochemical factories for the production of a variety of natural products, such as antibiotics, surfactants, and peptide toxins, following the thiotemplate model instead of the ribosomal route. Each NRPS module incorporates one monomer in the growing peptide chain in a directed manner by the concerted action of conserved domains catalyzing multistep reactions, thereby defining the chemical identity of the final product. An NRPS module comprises at least an adenylation (A) domain that specifically recognizes and activates a certain substrate, its cognate peptidyl carrier protein (PCP; also called T domain) and a condensation (C) domain that catalyzes the chain growth (for review, see Marahiel et al., 1997; Fischbach and Walsh, 2006; Koglin and Walsh, 2009). Beside these ‘core domains’ NRPS often contain ‘auxiliary domains,’ such as epimerization (E) or methylation (MT) domains, contributing to the structural diversity of natural peptide products (Walsh et al., 2001; Sieber and Marahiel, 2005). The genetic locus *ces* encoding the cereulide synthetase shows the typical architecture of NRPS gene clusters and comprises, beside the structural genes *cesA* and *cesB*, up- and downstream genes with known function in NRPS product assembly but also a putative hydrolase (*cesH*) of unknown function (**Figure 1**). The A domains of two out of the four modules in the structural genes *cesA* and *cesB* are interrupted by unusual insertions showing homologies to ketoreductases (KRs; Ehling-Schulz et al., 2005b, 2006a). Biochemical characterization of CesB1 revealed a novel logic for NRPS product assembly (Magarvey et al., 2006). These A domains recognize and select α-ketocarboxylic acids instead of α-hydroxy acids, which would be the expected precursors according to the toxin sequence. The newly described KR domain embedded in the A domains was shown to carry out chiral reduction on the toxin assembly line (Magarvey et al., 2006). A similar genetic architecture and modular organization with KR domains embedded in A domains was reported for NRPS catalyzing depsipeptides production in *Streptomyces* and cyanobacteria (Magarvey et al., 2006; Ding et al., 2011), thus suggesting that the chemosynthetic route of cereulide assembly is representative for other NRPS products with alternating ester and peptide bounds.

The order of modules in most NRPS systems is colinear with the product sequences, although it is increasingly becoming evident that the structural diversity of natural products does not always reinforce a strict collinearity (Wenzel and Müller, 2005; Lane and Moore, 2011). Indeed, a mass spectrometric screening and profiling approach of about 80 *B. cereus* strains demonstrated a high microheterogeneity of cereulide (Stark et al., 2013). In depth analysis of the emetic reference strain F4801/72 by UPLC-TOF MS and ion-trap MS^n^ revealed at least 18 cereulide variants that showed significant differences in their ionophoric properties and toxicity (Marxen et al., 2015a). Commonly, chemical variants of non-ribosomal peptide toxins found in other organisms, such as cyanobacteria, are based on the molecular diversity found within the structural genes of different strains or different species (Dittmann et al., 2013). In contrast, cereulide and its 18 variants are produced by a single NRPS synthetase (Ehling-Schulz et al., 2005b; 2006a) simultaneously in the same strain, interrogating the exact mode of toxin production. Just recently, a novel mechanism for the biosynthesis of cereulide was proposed, based on dipeptide rather than on single monomers as the basic modules in tetradepsipeptide assembly (Marxen et al., 2015b). The emetic strains analyzed so far produce all the same set of isocereulides, although at different concentrations. The contribution of the different cereulide variants to food intoxications caused by emetic *B. cereus* is hitherto unknown and further studies are needed to decipher the health risk posed by the different isocereulides. Such studies would also provide valuable information for framing and improving knowledge based risk assessments of *B. cereus* toxins in food production and processing.

### pCER270: A pXO1 like Megaplasmid Encoding the Cereulide NRPS

The *ces* cluster of emetic *B. cereus* is located on a 270 kb megaplasmid, named pCER270. The pCER270 shares its backbone with the toxin plasmid pXO1 of *B. anthracis* and the *ces* genes are inserted in a highly conserved part of these virulence plasmids (Ehling-Schulz et al., 2006a; 2011; Rasko et al., 2007). Although considerable progress has been made in recent years, the regulation of *ces* gene expression and cereulide production is far from being understood. It is increasingly becoming evident that cereulide formation is tightly linked to the metabolism of the producer strain by complex regulatory interactions between the toxin plasmid and the chromosome. The pCER270 plasmid carries about 10 regulators, including an ortholog of *abrB*, which might be involved in this interaction network and thus in various physiological processes. A key player in the embedment of cereulide toxin synthesis in the life cycle of emetic *B. cereus* is CodY, which is sensing the nutrient status of the cell (Frenzel et al., 2012; see next section for details).

### Megaplasmids and Pathotype Emergence

Likewise to *B. anthracis*, emetic *B. cereus* strains belong to highly clonal lineages in the rather panmictic *B. cereus* population. In contrast, the chromosomally encoded enterotoxins are distributed over the entire the *B. cereus* group (Prüss et al., 1999; Ehling-Schulz et al., 2005a; Guinebretiere et al., 2008; Didelot et al., 2009). Thus it is tempting to speculate that the toxin megaplasmids in the *B. cereus* group play a pivotal role in evolution of specific pathogenic traits. Very recently it has been shown that *B. cereus* group plasmids are vectors for duplicated non-essential chromosomal genes, which suggests that these plasmids may act as an evolutionary sink tank for emergence of novel eco- and pathotypes (Zheng et al., 2015). For instance, in the pCER270 the pXO1-PAI, bearing the anthrax toxin genes, is replaced by a 77 kb insertion that contains sporulation and germination genes, which may allow emetic strains to rapidly respond to changing environmental conditions such as accounted in foods (Ehling-Schulz et al., 2011). In addition to the Ces NRPS encoding mega plasmid, emetic strains can carry up to nine additional plasmids of variable size with yet unknown functions (Hoton et al., 2005; Ehling-Schulz et al., 2011). There are also some indications for the genetic transferability of the *ces* genes. In some rare cases the *ces* gene locus has been found in *Bacillus weihenstephanensis* (Thorsen et al., 2006; Hoton et al., 2009*)*, either located chromosomally or on a non-pXO1 like plasmid (Mei et al., 2014). In all emetic strains analyzed so far, mobile genetic elements (MGEs) are found in the proximities to the *ces* locus (Ehling-Schulz et al., 2011; Mei et al., 2014), which requires further attention. The contribution of these MGEs to the dynamics of progression of emetic *B. cereus* and their putative function in the emergence of novel eco- and pathotypes is hitherto unknown.

## Regulatory Circuits in Emetic *B. cereus*: Who are the Maestros that Orchestrate Metabolism and Virulence Development?

### Tight Temporal Control of *ces* Expression

**FIGURE 2:**
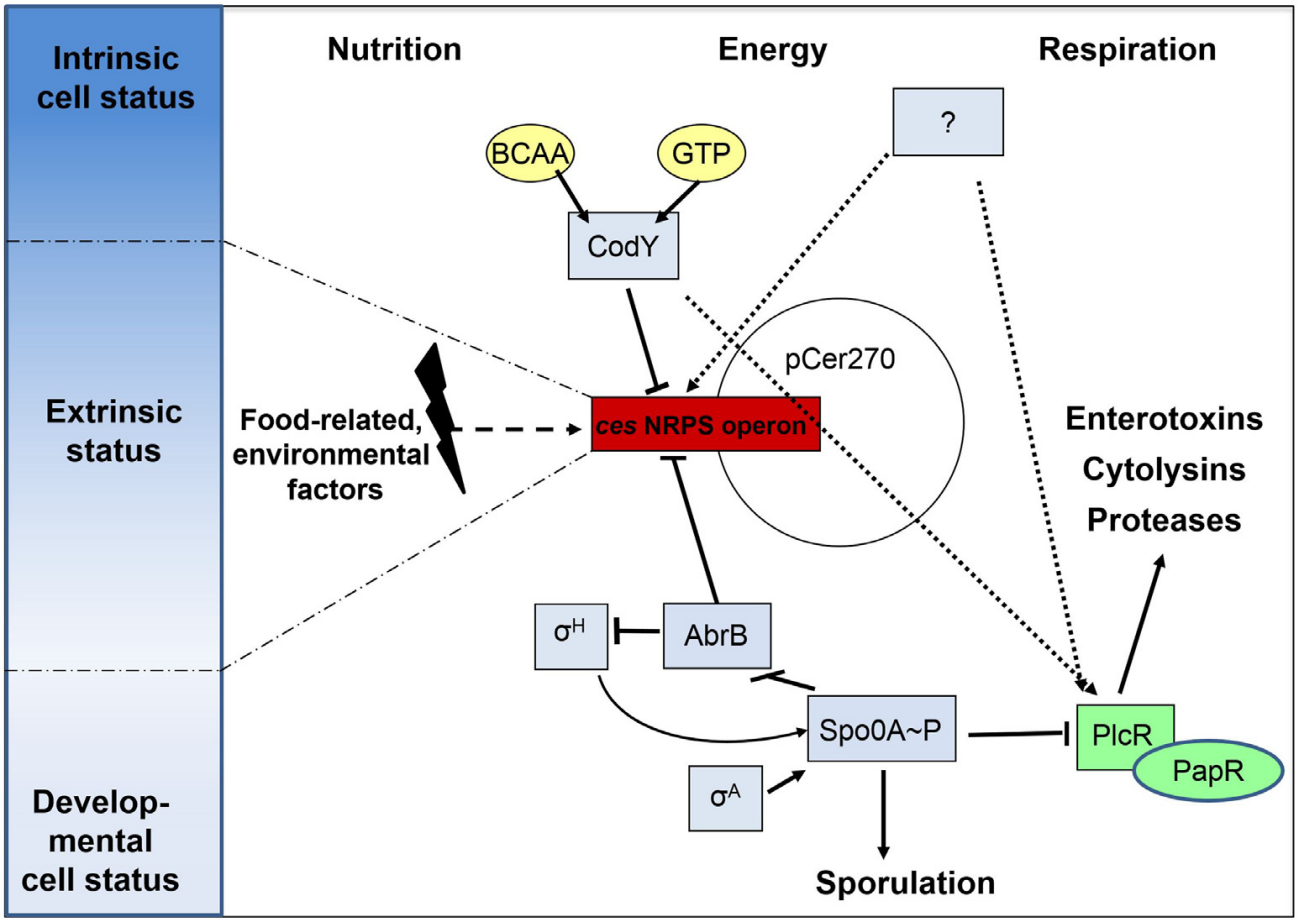
**Cereulide toxin synthesis is the result of a complex and multi-layered process.** Transcriptional regulation of the *ces* non-ribosomal peptide synthetase (NRPS) operon is tightly controlled by pleiotropic or metabolite-responsive transcriptional regulators such AbrB, Spo0A, and CodY. These ensure a correct timing of toxin synthesis within the cell cycle by integrating sporulation-inducing trigger factors and sensing the intrinsic nutritional and energy status of the cells by monitoring GTP and branched-chain amino acid (BCAA) levels. Although this ties cereulide formation to ceasing metabolic fitness levels, the unidirectional commitment to the developmental process of sporulation is induced later on by sigma factor H-dependent, self-reinforced levels of Spo0A-P, which are not affecting *ces* cluster transcription. Since cereulide formation significantly varies dependent on extrinsic stimuli, such as encountered in different food matrices, it is anticipated that additional transcriptional regulators could act on *ces* gene cluster expression. One prominent factor is oxygen availability, which might alike to CodY form a linker and an oppositely acting strategy for impinging the regulatory networks of virulence determination *via* PlcR/PapR and for that of cereulide formation. Solid arrows indicate a direct regulation while dashed arrows indicate an indirect regulatory effect.

Ces-NRPS gene transcription is initiated during late logarithmic growth, and rapidly switched off after the transition to the stationary phase (Dommel et al., 2011). This strict temporal regulation relies on the tight transcriptional control accomplished by transcriptional regulators that ensure the correct timing and integration of NRPS synthesis in the bacteria’s life cycle (**Figure 2**). Notable, the transcriptional regulators involved in *ces* synthetase regulation identified hitherto are all encoded chromosomally, while the *ces* gene cluster is located on a pXO1-like plasmid, pointing toward an intensive chromosomal – plasmid crosstalk and a tight link between virulence and metabolism. While several promoters were identified within the *ces* gene cluster, *cesP1* was shown to be the σ^A^-dependent main promoter, driving the polycistronic transcription of the *cesPTABCD* operon (Dommel et al., 2010; **Figure 1**). This promoter is not subjected to cell density responsive regulation by the PlcR-PapR QS system, but is directly repressed by the chromosomally encoded transition state regulator AbrB (Lücking et al., 2009).

Although cereulide synthesis is independent of later sporulation processes, it requires the early, housekeeping sigma factor A (σ^A^) promoted transcription of *spo0A*, the master regulator of sporulation in bacilli (Lücking et al., 2009). These initial, low levels of the phosphorylated form of Spo0A release AbrB repression by blocking *abrB* transcription (Strauch et al., 1990), which is reflected by the cereulide-deficient phenotype of a *B. cereus spo0A* null mutant (Lücking et al., 2009). The finding that complementation of the *spo0A* mutant with *spo0A* under the sole control of the sporulation-associated σ^H^-dependent promoter results in very low cereulide amounts, underpins that *ces* gene transcription is associated with an early and transient developmental state, when the level of phosphorylated Spo0A is too low to pinch the unidirectional sporulation process. The phosphorylation state of Spo0A is determined by the phosphate flux through a complex phosphorelay network built of several kinases, phosphotransferases, phosphatases, and kinase inhibitors, of which Spo0A is the final receiver protein (Hoch, 1993). In *B. subtilis*, this signal transduction pathway is fueled by autophosphorylating transmembrane histidine kinases (KinA–KinE), which respond to diverse environmental stresses and metabolic signals (LeDeaux et al., 1995). The identity of specific activating ligands is still elusive and controversially discussed (Tan and Ramamurthi, 2014; Devi et al., 2015). Additionally, the function of KinA–KinE related kinases has not been established in *B. cereus* yet. However, we could show that an external addition of long-chain polyphosphates (polyPs), which are widely used as additives in the food industry, negatively influences *ces* gene transcription and cereulide synthesis (see section below). This suggests that imbalances in the intracellular polyP/phosphate household could interfere with the phosphoryl flux through the relay, which results in altered Spo0A activity (Frenzel et al., 2011).

### CodY, the Major Conductor in Emetic *B. cereus* Pathogenesis

As early as with the first description of cereulide, it was apparent that the amino acid composition of the growth medium, in particular the amount of branched-chain amino acids (BCAAs), influences cereulide synthesis levels (Agata et al., 1999). This effect could be explained with the identification of CodY as one of the main regulators directly repressing *ces* gene transcription, while concomitantly exerting an indirect inducing effect on PlcR-dependent virulence factors (Frenzel et al., 2012). CodY is a pleiotropic transcriptional regulator that controls key metabolic intersections of the carbon and nitrogen metabolism and mediates stationary-phase adaptations in several low G+C Gram-positive bacteria (Sonenshein, 2007). The DNA-binding affinity of CodY is mediated by two types of ligand molecules, GTP and BCAA (Ratnayake-Lecamwasam et al., 2001; Shivers and Sonenshein, 2004). *In vivo* experiments with *B. cereus* AH187 showed that enhancing the endogenous CodY effector pool levels by external BCAA surplus feeding significantly reduced cereulide production (Frenzel et al., 2012). In this sense, CodY functions in *B. cereus* as an adaptor that mediates the regulatory crosstalk between chromosome and megaplasmid, and furthermore links the cell’s nutritional and energy status to pathogenicity. Cereulide synthesis is hence directly coupled to nutrient availability and *ces* gene expression takes place when levels of the CodY co-repressors pools decline, but the cell’s starvation is not so severe, that the sporulation pathway is already initiated (**Figure 2**). In this phase of the cell cycle, which is characterized by transient de-repression of CodY regulon members (Belitsky and Sonenshein, 2011; Brinsmade et al., 2014), the cell has to ensure an effective resource allocation and amino acid flow to achieve cellular maintenance, sustain growth, and to allow the energy-intensive formation of secondary molecules such as cereulide. Very logically, relieve of CodY-mediated repression concurrently leads to an enhanced synthesis of immune inhibitor (InhA) metalloproteases to maximize the usage of external nutrients (Frenzel et al., 2012). This indicates that a delicate intrinsic and extrinsic balance of amino acid levels is necessary to allow cereulide formation. The pool of amino acids must be sufficiently low to relieve *ces* promoter repression, while at the same time levels must be high enough to serve as direct precursors for the cereulide peptide. This is in excellent agreement with the observation at the food–bacteria interface: nutrient rich circumstances, especially proteinaceous food matrices, are less supportive for cereulide biosynthesis as are farinaceous foods (Agata et al., 2002; Dommel et al., 2010).

In opposite to its repressive effect on *ces* genes transcription, CodY promotes the expression of genes belonging to the PlcR virulence regulon in emetic strains (Frenzel et al., 2012; **Figure 2**). PlcR, the regulator of the major quorum sensing system in *B. cereus*, (Slamti and Lereclus, 2002), activates the transcription of a high number of secreted virulence genes encoding for enterotoxins, hemolysins, cytotoxins, proteases, or phospholipases (Gohar et al., 2008). Deletion of *codY* resulted in a phospholipase- and β-hemolytically inactive phenotype, which was linked to significantly lowered transcription of these genes (Frenzel et al., 2012). Since this phenotype was corroborated in studies with non-emetic strains (Lindbäck et al., 2012), CodY serves as a good example, how well-conserved chromosomal transcriptional regulators are adapted for feeding novel genetic material, such as the pCER270 megaplasmid, into ancient, or previously established, regulatory networks, as part of genetic diversification strategies.

However, gel-shift studies indicated that CodY is not a direct transcriptional activator of PlcR regulon genes, which implies that either an unknown regulator or posttranscriptional regulatory events are likely to mediate its effect (Frenzel et al., 2012). This dual-functioning nature of CodY on the *ces* genes and on the PlcR regulon could equip emetic strains with a powerful switching tool: when the intracellular levels of BCAAs and GTP is high, reflecting an extracellular milieu rich in proteins and energy sources as is found in animal host, CodY might mediate switches to the transcription of PlcR-dependent genes coding for proteases, phospholipases, enterotoxins, or hemolysins, concomitantly reducing the costly production of cereulide by the NRPS megaenzymes. *Vice versa*, ceasing amino acid availability that signals a necessity to enter diverging cellular differentiation processes might down regulate the extensive secretome production and thus allows a switch to cereulide production as a defensive strategy against competitors.

In summary, the decision to produce cereulide and PlcR regulon associated virulence factors is substantially mediated by the action of global transcriptional regulators. In emetic *B. cereus* strains, both virulence circuits are interconnected by the action of CodY and Spo0A. These, on the one hand, sense and respond to the cell’s intrinsic energy and metabolic status, and on the other hand act as timing devices and switching points for cellular differentiation processes, such as sporulation (**Figure 2**). However, this regulatory framework shows a certain degree of plasticity, which is reflected by the impact of various intrinsic and extrinsic stimuli that modulate the level of toxin production.

## Cereulide Synthesis is Shaped by a Complex Panoply of Environmental Cues

### External Signals

Cereulide production is linked to growth within certain, but not easily to define, limits. For instance, several studies provide evidence that the temperature range for growth of emetic *B. cereus* strains is broader than the temperature range in which cereulide production is actually detected (Finlay et al., 2000; Carlin et al., 2006; Apetroaie-Constantin et al., 2008). Besides temperature, the availability of oxygen plays a pivotal role in the formation of cereulide. Cereulide biosynthesis requires the presence of oxygen, though it is favored under low oxygen tensions (Finlay et al., 2000; Jääskeläinen et al., 2004). This indicates that channeling respiratory pathways and also the regulation of the redox state of the cell, which has been linked to enterotoxin synthesis (Duport et al., 2006), may also be important for cereulide formation. However, there have been contradicting findings concerning the impact of oxygen on cereulide synthesis at the food–bacteria interface (Agata et al., 2002; Rajkovic et al., 2006; Shaheen et al., 2006), which might be explained by the fact that a plethora of additional stimuli, as such are encountered in the complex nutrient environment, play another determinative role in toxin synthesis. A recent broad-scale, long-term study on cereulide production potentials in food matrices and on cereulide intoxication cases provided deeper insights on the prerequisites for cereulide production in foods (Messelhäusser et al., 2014). This study revealed that a complex interplay of fatty acids, C-sources, N-sources, micro- and macro-nutritional environments in combination with global factors such as pH and water availability determines the risk of food-borne intoxications, either by stimulating or inhibiting cereulide synthesis. For instance, it has been noticed that the ratio of external glycine, Na^+^ and K^+^ ions also has an impact on the level of cereulide production (Apetroaie-Constantin et al., 2008). According to our current knowledge on cereulide as being a K^+^ ionophore, it is tempting to speculate that the K^+^ homeostasis might be a driving force in cereulide production.

### Integration of External Signals

**FIGURE 3:**
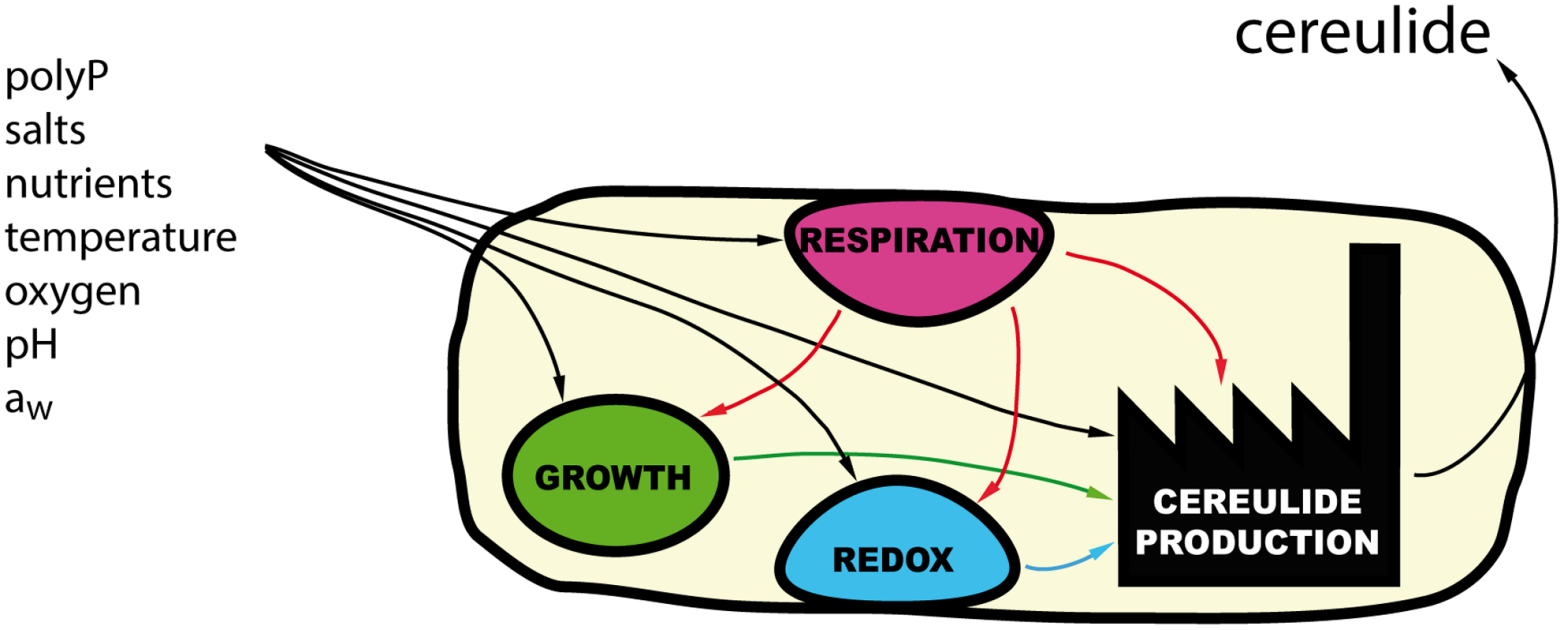
**Environmental signals and cereulide production.** An array of external signals, acting on various physiological processes such as respiration, redox potential regulation, or growth, are integrated by the bacterium and modulate cereulide production.

Nonetheless, how these external signals are perceived by the cell and transduced to the *ces* promoter is unknown. Additionally, how those external cues modulate the cell’s physiology to induce combinatory or opposite stimulating or inhibitory effects on cereulide synthesis, is a very complex question that still awaits elucidation. The conditions permitting cereulide synthesis are far more stringent than the once allowing growth of *B. cereus*, indicating that specific trigger factors and/or combinations thereof are impacting cereulide production (**Figure 3**). For instance, increasing sodium chloride concentrations in liquid medium lowers cereulide production at the transcriptional and toxin level, although the growth rate of strains is not affected, rendering it unlikely that the mechanism can be simply related to salt stress (Dommel et al., 2011). A similar observation was made concerning the influence of widely used food additives, long chain polyPs, which are cation-sequestering straight-chain polymers of condensed orthophosphoric acid residues (Frenzel et al., 2011). While treated with non-lytic, non-bacteriostatic polyP concentrations, *B. cereus* growth rates in liquid culture, or final cell numbers in a model food were not significantly altered. However, polyPs interfered with early stages of the toxin formation process and significantly delayed and reduced *ces* promoter activity, *ces* gene transcription, and hence, quenched cereulide synthesis. How external polyP addition affects the intracellular physiology is unclear to date. *B. cereus*, like many other bacteria, itself endogenously synthesizes and hydrolyzes natural polyP analogs that fulfill manifold physiological functions, ranging from metabolic regulation to protein phosphorylation (Shi et al., 2004; Rao et al., 2009). Thus, external polyP addition obviously leads to imbalances in the internal polyP/Pi ratio, which might not only affect the sporulation process, but also catalytic processes of the NRPS modules.

Though some substances, including bacteriocins, organic acids, and nisin, have been reported to inhibit the growth of *B. cereus* in the food environment (Valero and Salmeron, 2003; Galvez et al., 2007; Ter Beek and Brul, 2010), these studies did not focus on the targeted inhibition of cereulide or enterotoxin formation. Therefore, it is unclear to date, whether other food additives besides polyPs can directly affect virulence regulation networks in *B. cereus.* Nevertheless, the example of polyPs shows that understanding of how external toxin trigger factors are perceived and integrated in the physiological and metabolic response of *B. cereus* could pave the way for tailored hurdle concepts in food production and food processing chains.

## A Home for Emetic Strains: Seeking for their Natural Habitat

### Megaplasmids, Key Determinants of the Ecological Niche for *B. cereus*

Non-emetic and emetic *B. cereus* occupy a broad and, especially for emetic strains, ill-defined ecological niche. *B. cereus* is present in the soil, in the gut of insects and of mammals, on plant roots (Jensen et al., 2003; Ceuppens et al., 2013) or on mycorrhizae-associated fungi (Smith et al., 1999; Toljander et al., 2006; Lakshmanan et al., 2012). Although many *B. cereus* isolates have been sequenced, strain- or subgroup-specific genetic signatures displaying possible adaptation events to each of these environments could not be identified yet (Ehling-Schulz and Messelhäusser, 2014). In contrast, the ecological niches of *B. thuringiensis* and *B. anthracis*, two species genetically close to *B. cereus*, are far better studied and it is apparent that their lifestyle is defined and shaped by their plasmid-encoded genetic material. *B. thuringiensis*, which is characterized by expressing Cry toxins *via* large plasmids, is an entomopathogen, while *B. anthracis*, carrying the pXO1 and pXO2 mega plasmids with the three-partite Anthrax exotoxin, is a high-potential mammal pathogen. Alike to *B. thuringiensis* and *B. anthracis*, the emetic subgroup of *B. cereus* strains carries the cereulide NRPS cluster encoding megaplasmid pCER270. However, although cereulide seems to act as an effector of ecological competition against a range of Gram-positive bacteria and some fungi (Ladeuze et al., 2011; Tempelaars et al., 2011), it is still unclear, why cereulide producers are frequently associated with human intoxication cases stemming from cereulide production in food matrices.

Since human infections by emetic strains are rare, mammals are probably not the primary target and not the preferred host to thrive and feed on. Interestingly, the prevalence of emetic strains was found to be low in environmental samples, but high in food materials (**Table 1**). Whereas a high number of soil samples contain *B. cereus* or *B. thuringiensis* strains, very few of these strains were emetic, and no emetic strains were found amongst isolates recovered from invertebrates samples. Furthermore, emetic strains were detected in the intestine of small root-, seed-, and insect-consuming mammals, representing as much as 16% of *B. cereus* isolates, but not in the feces of herbivorous mammals (**Table 1**). By contrast, the proportion of emetic strains within *B. cereus* isolates ranged from 1.4 to 10.2% in food samples obtained from retail food shops (random food), and this proportion increased to 17–32% in samples involved in food poisoning cases (non-random food; see **Table 1**). The higher prevalence of emetic strains in intoxication cases-associated food, compared to randomly sampled food, likely reflects the increased awareness of cereulide intoxications by food authorities and/or diagnostic laboratories and is supported by an optimized screening toolbox for emetic strains *via* qPCR and cereulide toxin detection *per se* (Ehling-Schulz and Messelhäusser, 2013). However, the true incidence of emetic strains might still be underestimated since emetic strains are only weakly or non-hemolytic (Ehling-Schulz et al., 2005a; Pirhonen et al., 2005) and thus might be misdiagnosed as being not *B. cereus*. For instance, in some studies on *B. cereus* prevalence in food, non-hemolytic strains were discarded and emetic strains were found to be absent or extremely rare (Rosenquist et al., 2005; Ankolekar et al., 2009; Samapundo et al., 2011).

**Table 1:** Prevalence of emetic *Bacillus cereus* strains in samples of various origin.

Sample origin	*n*	Bc	Bce %	Reference
Soil	nd	543	0.2	Hoton et al. (2009)
Soil	80	61	0.0	Altayar and Sutherland (2006)
Soil	nd	38^a^	0.0	Forghani et al. (2014)
Soil	15	12	0.0	Santos et al. (2011)
Invertebrates	nd	58	0.0	Hoton et al. (2009)
Shrew or vole intestine	nd	109	16.5	Hoton et al. (2009)
Cow or horse feces	66	nd	0.0	Altayar and Sutherland, 2006)
Food (random)	nd	582	1.4	Hoton et al. (2009)
Food (random)	742	402	10.2	Messelhäusser et al. (2014)
Food (non-random)	nd	95	31.6	Hoton et al. (2009)
Food (non-random)	3654	187	17.1	Messelhäusser et al. (2014)

*n, number of samples; Bc, number of *B. cereus* strains isolated from the samples; Bce %, proportion of emetic *B. cereus* strains within all *B. cereus* isolates. ^a^*B. cereus* and *B. thuringiensis* strains.*

### Entrance of Emetic Strains to Food and Food Production Processes

A literature review on the association of the emetic strains with different food components reveals a rather heterogeneous distribution of these strains (**Table 2**). For instance, emetic strains seem to be rare in meat products, but are occasionally present in potato, rice, mushrooms, or dairy products with such a high prevalence that even out-competes accompanying bacteria. It might be speculated that certain food niches provide an advantageous environment for growth/and or antibiotic production, allowing a selective substrate occupancy by cereulide producers. Elevated temperatures commonly used during food processing might further select for emetic strains, which show an increased heat resistance with respect to vegetative growth and spore D_10_-values compared to non-emetic strains (Carlin et al., 2006). Thus, the entrance routes into the food chain as well as the modes of selective enrichment of emetic strains within foodstuff are becoming a hotspot of interest during recent years.

**Table 2:** Prevalence of *B. cereus* emetic strains in food samples.

Sample origin	*n*	Bc	Bce %	Reference
Broccoli (raw)	25	8	0.0	Flores-Urban et al. (2014)
Carrot (raw)	25	21	0.0	Flores-Urban et al. (2014)
Carrot (raw)	25	22	0.0	Altayar and Sutherland, 2006)
Potato (raw)	25	21	16.0	Altayar and Sutherland, 2006)
Potato (raw)	nd	8	60.0	Hoornstra et al. (2013)
Coriander (raw)	25	11	0.0	Flores-Urban et al. (2014)
Lettuce (raw)	25	17	0.0	Flores-Urban et al. (2014)
Lettuce (raw)	48	44	0.0	Messelhäusser et al. (2014)
Rice (boiled)	54	41	17.1	Delbrassinne et al. (2012)
Rice (raw)	nd	103^a^	26.2	Forghani et al. (2014)
Rice (raw)	78	44	11.3	Messelhäusser et al. (2014)
Fruits	27	15	0.0	Messelhäusser et al. (2014)
Dried mushrooms	135	110	10.0	Messelhäusser et al. (2014)
Dairy products	212	119	18.5	Messelhäusser et al. (2014)
Dairy products	809	508	4.7	Messelhäusser et al. (2010)
Dairy products	nd	161	10.0	Wijnands et al. (2006)
Meat products	121	0	0.0	Messelhäusser et al. (2014)
Meat products	nd	24	0.0	Wijnands et al. (2006)

*n, number of samples; Bc, number of *B. cereus* strains isolated from the samples; Bce %, proportion of emetic *B. cereus* strains within all *B. cereus* group isolates. ^a^*B. cereus* and *B. thuringiensis* strains.*

### Potential Ecological Niches of Emetic Strains

One environmental route for gaining entry into the food chain might be associated with *B. cereus* thriving in the intestine of small rodents. These feed on plant roots or on invertebrates, which in turn feed on plant roots and tubers. Upon harvest, the root microflora contaminates the crop. In foodborne outbreaks, the incriminated food often contains farinaceous foods, such as rice (Gilbert and Parry, 1977) or pasta (Mahler et al., 1997; Dierick et al., 2005; Naranjo et al., 2011). Emetic strains appear to be preferentially present in specific plants, and their occurrence in dairy products might be a consequence of milk contamination. A survey of *B. cereus* strains isolated from rice paddy fields showed that 44% of these isolates were capable of producing the emetic toxin (Ueda and Kuwabara, 1993), and emetic strains were found in the extracellular space of potato tubers, behaving as endophytic bacteria (Hoornstra et al., 2013). Emetic strains might be innocuous to potato tubers, because in contrast to most *B. cereus* strains, they cannot degrade starch and differ in their biofilm production capacities (Ehling-Schulz et al., 2005a; Auger et al., 2009). An endophytic lifestyle of emetic strains in starch-rich plants would explain, why these strains are often found in starch-rich foods (e.g., pasta, rice), despite their inability to hydrolyze starch.

It was postulated that cereulide might provide an adaptive advantage to emetic strains by scavenging potassium within the endophytic and soil-associated environments (Teplova et al., 2006; Ekman et al., 2012) where potassium is rare (Hoornstra et al., 2013). Cereulide might also protect the tuber against attacks by pathogenic fungi by its antifungal activity (Ladeuze et al., 2011), possibly indicating a symbiotic or synergistic effect of emetic strains and plants. Therefore, it seems more reasonable that enrichment of cereulide in certain foods (Dommel et al., 2010; Messelhäusser et al., 2014) might be the consequence of dysregulated cereulide production in non-natural environments, such as encountered in some food matrices, rather than posing an ecological or evolutionary meaning to *B. cereus* in first instance.

**FIGURE 4:**
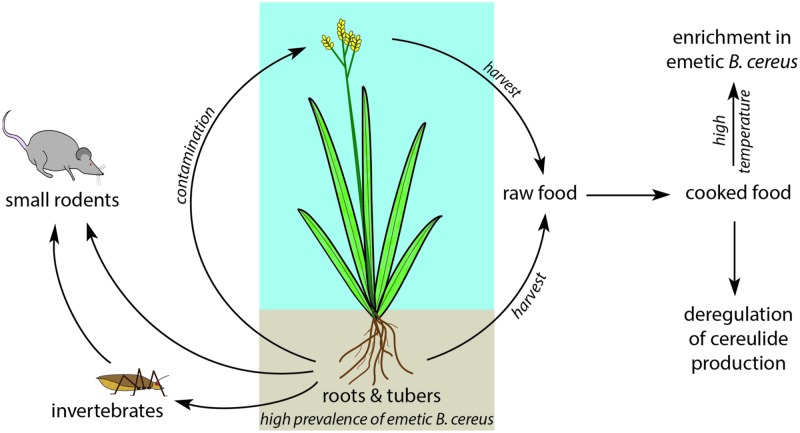
**Hypothetical ecological niche of *Bacillus cereus* emetic strains.** Emetic strains of *B. cereus* are likely to colonize roots and tubers of plants, either as endophytic symbionts or as biofilms. Aerial parts of the plants might then be contaminated by these strains, upon harvest or by small animals feeding on the underground plants parts. The aerial parts of the plants can also, when used as fodder, contaminate cow udder and therefore milk. Since emetic strains spores are more thermoresistant than other *B. cereus* strains spore, cooked food will then be enriched in emetic strains which, in this unnatural environment, could overproduce the emetic toxin.

Summarizing the current knowledge on emetic strains, it seems reasonable that they differ concerning their preferred ecological niches compared to their non-emetic siblings. The primary natural habitat of emetic strains might therefore be linked to roots, tubers and mycorrhizae of some plants (**Figure 4**). Thus, the extremely potent human toxin cereulide might be a key element for their adaptation to the natural (soil) environments and might be a key requisite to thrive in specified niches.

## Future Perspectives

An in-depth understanding of the molecular mechanisms involved in the specialization of emetic strains for food product colonization is a prerequisite to develop novel prevention and effective counteraction strategies. Toward this end, deciphering the ecological function of the cereulide toxin would be an important step and could also guide efforts to uncover yet unknown environmental sources of emetic strains. Recent reports point toward plants as a potential source for emetic strains, although the nature of this association and its potential contribution to food contamination is hitherto unknown.

In addition, further research is necessary to elucidate the function of the intensive plasmid–chromosomal cross talk for the microevolution and emergence of novel pathotypes in *B. cereus sensu lato*. The regulatory plasmid – chromosomal interactions are apparently much more pronounced within the *B. cereus* group than in *B. subtilis*, underpinning that a simple comparison and/or deduction of the function and regulons of transcriptional regulators is not possible between *B. cereus* and *B. subtilis* strains. Results from recent genome studies indicate that the inter-plasmid–chromosomal gene duplications play a pivotal role in intraspecies diversification of *B. cereus* and it is tempting to speculate that it also plays an important role in the emergence of specific pathogenic traits.

## Conflict of Interest Statement

The authors declare that the research was conducted in the absence of any commercial or financial relationships that could be construed as a potential conflict of interest.
